# Delayed NLRP3 inflammasome inhibition ameliorates subacute stroke progression in mice

**DOI:** 10.1186/s12974-022-02674-w

**Published:** 2023-01-04

**Authors:** Maximilian Bellut, Michael Bieber, Peter Kraft, Alexander N. R. Weber, Guido Stoll, Michael K. Schuhmann

**Affiliations:** 1 Department of Neurology, University Hospital Würzburg, Josef-Schneider-Straße 11, 97080 Würzburg, Germany; 2 Department of Neurology, Klinikum Main-Spessart Lohr, Lohr, Germany; 3 Department of Immunology, Interfaculty Institute of Cell Biology, University of Tübingen, Tübingen, Germany; 4 iFIT-Clusters of Excellence EXC 2180 “Image-Guided and Functionally Instructed Tumor Therapies” and EXC 2124 “Controlling Microbes to Fight Infection”, University of Tübingen, Tübingen, Germany

**Keywords:** Ischemic stroke, Inflammasome, NLRP3, Middle cerebral artery occlusion, Neuroinflammation, Secondary infarct growth

## Abstract

**Background:**

Ischemic stroke immediately evokes a strong neuro-inflammatory response within the vascular compartment, which contributes to primary infarct development under vessel occlusion as well as further infarct growth despite recanalization, referred to as ischemia/reperfusion injury. Later, in the subacute phase of stroke (beyond day 1 after recanalization), further inflammatory processes within the brain parenchyma follow. Whether this second wave of parenchymal inflammation contributes to an additional/secondary increase in infarct volumes and bears the potential to be pharmacologically targeted remains elusive. We addressed the role of the NLR-family pyrin domain-containing protein 3 (NLRP3) inflammasome in the subacute phase of ischemic stroke.

**Methods:**

Focal cerebral ischemia was induced in C57Bl/6 mice by a 30-min transient middle cerebral artery occlusion (tMCAO). Animals were treated with the NLRP3 inhibitor MCC950 therapeutically 24 h after or prophylactically before tMCAO. Stroke outcome, including infarct size and functional deficits as well as the local inflammatory response, was assessed on day 7 after tMCAO.

**Results:**

Infarct sizes on day 7 after tMCAO decreased about 35% after delayed and about 60% after prophylactic NLRP3 inhibition compared to vehicle. Functionally, pharmacological inhibition of NLRP3 mitigated the local inflammatory response in the ischemic brain as indicated by reduction of infiltrating immune cells and reactive astrogliosis.

**Conclusions:**

Our results demonstrate that the NLRP3 inflammasome continues to drive neuroinflammation within the subacute stroke phase. NLRP3 inflammasome inhibition leads to a better long-term outcome—even when administered with a delay of 1 day after stroke induction, indicating ongoing inflammation-driven infarct progression. These findings may pave the way for eagerly awaited delayed treatment options in ischemic stroke.

**Supplementary Information:**

The online version contains supplementary material available at 10.1186/s12974-022-02674-w.

## Background

Cerebral ischemia elicits a strong neuroinflammatory response which occurs in a strict spatio-temporal order [1]. During the first 24 h inflammation is mainly restricted to the intravascular compartment [2], probably driven by local platelet and leukocyte derived danger-associated molecular patterns (DAMPs) and chemokines [3, 4]. A second inflammatory wave is induced by neuronal and glial cell death with ensuing infiltration of immune cells into the brain parenchyma [5, 6]. Dying neurons and glial cells are an important additional source of DAMPs [7, 8]. DAMPs induce the inflammasome [9]. The inflammasome, in turn, modulates a wide range of downstream inflammatory responses [10]. While it is well-established that early interference with inflammation prevents ischemia–reperfusion (I/R) injury in transient middle cerebral artery occlusion (tMCAO) during the first 24 h after recanalization [11], it is largely unknown, whether delayed parenchymal neuroinflammation is amenable to treatment. Anti-inflammatory treatment options beyond the classical limited time window for recanalization are of outmost clinical importance. To address this issue, we focused on the long-term outcome after inhibition of the NLR family pyrin domain containing 3 (NLRP3) inflammasome, that has recently been proven to play a central role in the control of inflammatory mechanisms in stroke during I/R injury [11, 12].

NLRP3 is responsible for the autoactivation of Caspase 1 and the subsequent cleavage and secretion of the pro-inflammatory cytokines Interleukin (IL) 1β and IL18 as well as the induction of pyroptosis via gasdermin D cleavage [13, 14]. Pyroptosis in turn leads to a further release of inflammatory DAMPs [15].

Lately, we have shown an upregulation of NLRP3 and its downstream pro-inflammatory cytokines during the early phases of ischemic stroke (IS). Functionally, immediate treatment with the NLRP3-specific inhibitor MCC950 ameliorated infarct growth during I/R and improved functional outcome within the first 24 h after stroke onset [11, 12]. We here examined whether delayed parenchymal neuroinflammation contributes to subacute infarct expansion beyond 24 h and if the latter can be diminished by NLRP3 inhibition.

## Materials and methods

### Materials

10 mg of the inflammasome-inhibitor MCC950 (#538120, Merck, Darmstadt, Germany) were dissolved in 1 ml of sterile water and further diluted with PBS to the final concentration of 5.5 mg/ml or 50 mg/kg body weight (BW), respectively. PBS, sterile water, anti-NeuN (MAB377) and anti-β-Actin (A5441) were all purchased by Merck. TRIzol reagent (#15596026), TE buffer (#12090015), TaqMan Reverse Transcription Reagents (#N8080234), random hexamer primers (#SO142), the rtPCR primers for Caspase 1 (Csp1, Mm00438023_m1, #4331182), Interleukin 1β (Il1b, Mm00434228_m1. #4331182), Interleukin 18 (Il18, Mm00434225_m1, #4331182) as well as glyceraldehyde 3-phosphate dehydrogenase (GAPDH; TaqMan Predeveloped Assay Reagents for gene expression, #4352339E), ProLong Gold Antifade Mountant with DAPI dye (#P36931) and as secondary antibodies Alexa FluorTM 488 goat anti-mouse IgG (A11001), 488 donkey anti-rabbit IgG (A21206), 647 goat anti-rat IgG (A21247), 546 goat anti-rabbit IgG (A11035) and 488 donkey anti-rat IgG (A21208,) were provided by Thermo Fisher Scientific (Waltham, MA, USA). Anti-NLRP3 (AG-20B-0014) was purchased by Adipogen Life Sciences (San Diego, CA, USA). Anti-CD4 (#100506), and anti-Ly6G (#127636) were obtained by BioLegend (San Diego, CA, USA). Anti-GFAP (ab7260), anti-MAP2 (ab32454) and anti-Iba1 (ab178846) were produced by Abcam (Cambridge, UK). Peroxidase AffiniPure Donkey Anti-Mouse IgG (#715–035-150) was delivered by Jackson ImmunoResearch (Cambridge, UK) (see also Additional file 1: Tables S1,  S2).

### Animals and sample size calculation

We used 6–8-week-old male C57Bl/6 N mice, purchased from Charles River Laboratories (Sulzfeld, Germany). In all, 53 mice were used for this study. 10 mice were assigned to a 24 h post-tMCAO treatment group (MCC950 d1), 9 mice were assigned to a prophylactic pre-tMCAO treatment group (MCC950 d0), and 10 mice to the respective vehicle groups each. Each of the aforementioned groups passed a 7 day long reperfusion phase. Since the two vehicle groups did not differ regarding mortality (Additional file 1: Fig. S1), infarct size and clinical tests, the surviving animals in the vehicle groups were pooled to reduce the total number of necessary animals best possibly according to the 3R-principle. Moreover, 14 mice were used as sham and vehicle groups for a post 24 h reperfusion study. After randomization, tMCAO was conducted for 30 min. Surgery and evaluation of all readout parameters were performed blinded to the experimental groups. Assuming a reduction of infarct volume of 30% as functionally relevant and a standard deviation of 20% to the respective mean values, a group size of ≥ 8 was necessary to show this effect with a power of 0.8 and a probability of a type I error of < 0.5 (calculated with GraphPad StatMate 2.00). All in all, 27 mice survived the 7 day observation period (11 in the pooled vehicle group, 8 in the MCC950 d0 group, 8 in the MCC950 d1 group) and were used for infarct size measurement and clinical tests. Out of these, 9 mice of the vehicle group, 7 of the MCC950 d0 group and 7 of the MCC950 d1 group were used for further immunohistological and Western Blot analysis. 14 mice (7 vehicle and 7 sham) were used for Western Blot analysis after only 24 h of reperfusion.

### Animal treatment

Animals were treated with 100 μl of the inflammasome-inhibitor MCC950 (50 mg/kg BW) or the same volume of vehicle (1 × PBS), which were administered by an intraperitoneal injection 24 h after tMCAO (MCC950 d1 group) or directly before occluding the MCA for 30 min (MCC950 d0 group) [11].

### Ischemia model

The tMCAO model was used to induce focal cerebral ischemia as described in detail before [16]. The experiments were carried out blinded. An independent researcher who was not involved in data analysis coded and randomized the mice. To reduce the variability of our outcome parameters caused by sex-differences only male mice were used in the study [17]. Before tMCAO the mice were anesthetized with 2% isoflurane. 200 mg/kg BW metamizol was injected subcutaneously and 4% lidocaine gel applied on the wound margins as analgesia. With a servo-controlled heating blanket, a body core temperature close to 37 °C was maintained throughout surgery. After a midline neck incision, a standardized silicon rubber-coated 6.0 nylon monofilament (6023910PK10; Doccol, Sharon, MA, USA) was inserted into the right common carotid artery and advanced via the internal carotid artery to occlude the origin of the MCA. After 30 min, the mice were re-anesthetized and the occluding filament removed to allow reperfusion. To reduce infarct variability all mice were operated by the same operator. Operation time did not exceed 15 min. The observation period accounted for 7 days. Sham mice were treated like mice of the vehicle group but the filament was removed directly after its insertion.

### Stroke vol﻿ume 

Animals were killed 7 days after tMCAO and the brains were cut in three 2 mm-thick coronal sections. The slices were stained for 20 min at 37 °C with 2% TTC to visualize the infarctions. Edema-corrected infarct volumes were calculated by planimetry (ImageJ software, National Institutes of Health, Bethesda, MD, USA) [18]. Moreover, one additional 10 µm thick slice, obtained out of the middle coronal section, was also analyzed histologically by MAP2 staining to depict neuronal damage as described previously [19].

### Assessment of functional outcome

The global neurological deficits were quantified conducting the Neuro Score, composed of the sum of the inverted Bederson score as well as the grip test. They were assessed after anesthesia abated at the day of the tMCAO experiments (day 0) as well as every 24 h until day 7 after stroke induction [20, 21]. Furthermore, the latency to move test, which measures the time a mouse needs to move one body length, and the modified Neurological Severity Scores (mNSS), a composite of motor, sensory, reflex and balance tests resulting in a deficit score from 0 (normal function) to 18 (maximal deficit), were assessed at the same timepoints [22, 23]. The clinical assessment at days 1–24 h post-tMCAO was performed directly before the treatment in the MCC950 d1 group took place.

### Exclusion criteria

Mice were excluded from endpoint analyses due to: (1) death before the predefined experimental endpoint; (2) Bederson score = 5 (at any timepoint after tMCAO); (3) weight loss > 20% at any timepoint after tMCAO. All in all, 3 mice were excluded due to death during the surgery and 2 due to weight loss > 20%.

### Quantitative real-time polymerase chain reaction (PCR)

After sacrification of the animals, we separated the cortices and basal ganglia tissue from both hemispheres for RNA isolation. Next to homogenization with TRIzol Reagent (1 ml per 100 mg tissue), chlorophorm was added and samples were centrifuged at 12,000 *g* for 5 min at 4 °C. The upper aqueous phase was collected and mixed with isopropyl alcohol for RNA precipitation, washed, dissolved in TE buffer and finally quantified spectrophotometrically. 1 µg of total RNA were used for reverse transcription with the TaqMan Reverse Transcription Reagents according to the manufacturer’s protocol using random hexamers. Primers for IL1β, IL18 and Caspase 1 were utilized. GAPDH was used as endogenous control. PCR was performed with equal amounts of cyclic deoxyribonucleic acid and water control in the StepOnePlus™ Real-Time PCR System (Applied Biosystems) using the TaqMan Universal 2X PCR Master Mix (Applied Biosystems). Each sample was measured in duplicate and all data points examined for integrity by analysis of the amplification plot. The comparative cycle threshold method was used for relative quantification of gene expression as described elsewhere [24].

### Protein extraction and Western Blot analysis

Protein extraction and Western blot analysis were performed according to standard procedures as previously described [25]. Therefore, dissected cortices and basal ganglia from the mouse brains were homogenized with RIPA buffer (25 mM Tris pH 7.4, 150 mM NaCl, 1% NP-40, 0.1% SDS) containing 4% proteinase inhibitor (cOmpleteTM protease inhibitor cocktail, Thermo Fisher Scientific) and sonified for 10 s. After centrifugation at 15,000 *g* for 30 min at 4 °C supernatants were used for bicinchoninic acid protein assay and subsequent Western Blot analysis. The lysates were mixed with 2 × SDS–PAGE loading buffer (final concentration: 60 mM Tris pH 6.8, 10% beta-mercaptoethanol, 5% SDS, 10% glycerol) at 95 °C for 10 min. 20 µg of total protein was loaded on the gel, electrophoresed and transferred to a nitrocellulose membrane. After blocking for 30 min with blocking buffer (5% nonfat dry milk, PBS, 0.05% Tween-20) membranes were incubated with the primary antibodies anti-GFAP (1:10.000), anti-NLRP3 (1:500), anti-Iba1 (1:500) and anti-actin mAb (1:1.000.000) at 4 °C overnight. As secondary antibodies Peroxidase AffiniPure Donkey Anti-Mouse IgG or Goat Anti-Rabbit IgG (1:2000) were used.

### Histology and immunohistochemistry

For histology brain tissue was cut in 2-mm-thick coronal sections, embedded in Tissue-Tek OCT compound and frozen. Brain sections were cut on a cryostat into 10-μm thin slices and used for all analysis. For immunohistochemistry, slices were post-fixated with ice-cold 100% methanol. Immunohistochemistry was performed according to standard procedures [26]. Mouse brains were stained for DAPI and with anti-GFAP (1:100), anti-NeuN (1:100), anti-CD4 (1:100), anti-Iba1 (1:100), anti-Ly.6 g (1:50) and anti-NLRP3 (1:100). Secondary antibodies were used in a dilution of 1:100. For measurement of the NeuN intensity, images at the level of the basal ganglia (0.4, 0.45 and 0.5 mm anterior from bregma) of 7–9 different animals for each group were recorded with a microscope (Leica DMi8 equipped with the DMC 2900/DFC 3000G camera control and LAS X software (Leica, Wetzlar, Germany)). Subsequently, after converting the images into 16-bit black and white files, the intensity of the NeuN staining was compared between the ipsilesional and contralesional hemispheres with ImageJ Analysis Software 1.52a (National Institutes of Health, Bethesda, MD, USA). For this purpose, the ratio of the overall intensity scores of the ipsilesional and contralesional hemisphere was calculated and served as a comparison parameter among each single animal. Within the same three brain sections per animal, NeuN counting was performed directly under the microscope in all together 10 priorly defined brain regions as outlined in the respective figure. The countings of CD4, Iba1 and Ly.6 g positive cells were performed within 5 ipsilesional and 5 contralesional priorly defined specific segments (in each case 3 cortical and 2 basal ganglial segments). The recording took place with the aforementioned microscope. The cells within the segments were counted manually.

### Statistical analysis

The results of the neurological tests are presented as bar graphs with the mean and the standard error of the mean (SEM) (latency to move test) or inter quartile range (IQR) (Neuro Score and mNSS). All the other results are presented as box plots indicating median, the 25th percentile, the 75th percentile, minimum and maximum. For statistical analysis the GraphPad Prism 8 software was used. Data were tested for Gaussian distribution with the D’Agostino-Pearson omnibus normality test and then analyzed by one-way analysis of variance (ANOVA) and in the case of repeated measurements two-way ANOVA with post hoc Tukey adjustment for *p* values or unpaired *t* test. Probability values < 0.05 were considered to indicate statistically significant results. A Log-rank (Mantel–Cox) test was used for the survival curves.

## Results

**Fig. 1:**
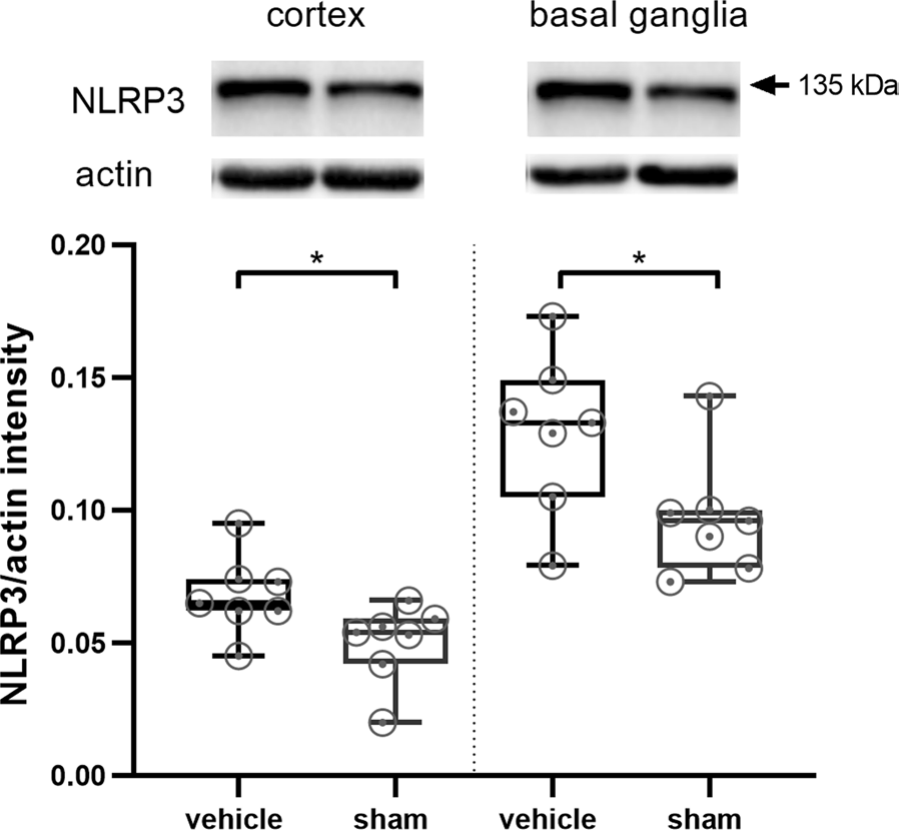
tMCAO leads to higher NLRP3 levels 24 h after stroke induction compared to sham mice. NLRP3 protein expression in cortex and basal ganglia of vehicle- and sham treated mice on day 1 after tMCAO. For densitometric quantification actin was used as a loading control (*n* ≥ 7). Data was analyzed by unpaired *t* test. *p < 0.05

**Fig. 2:**
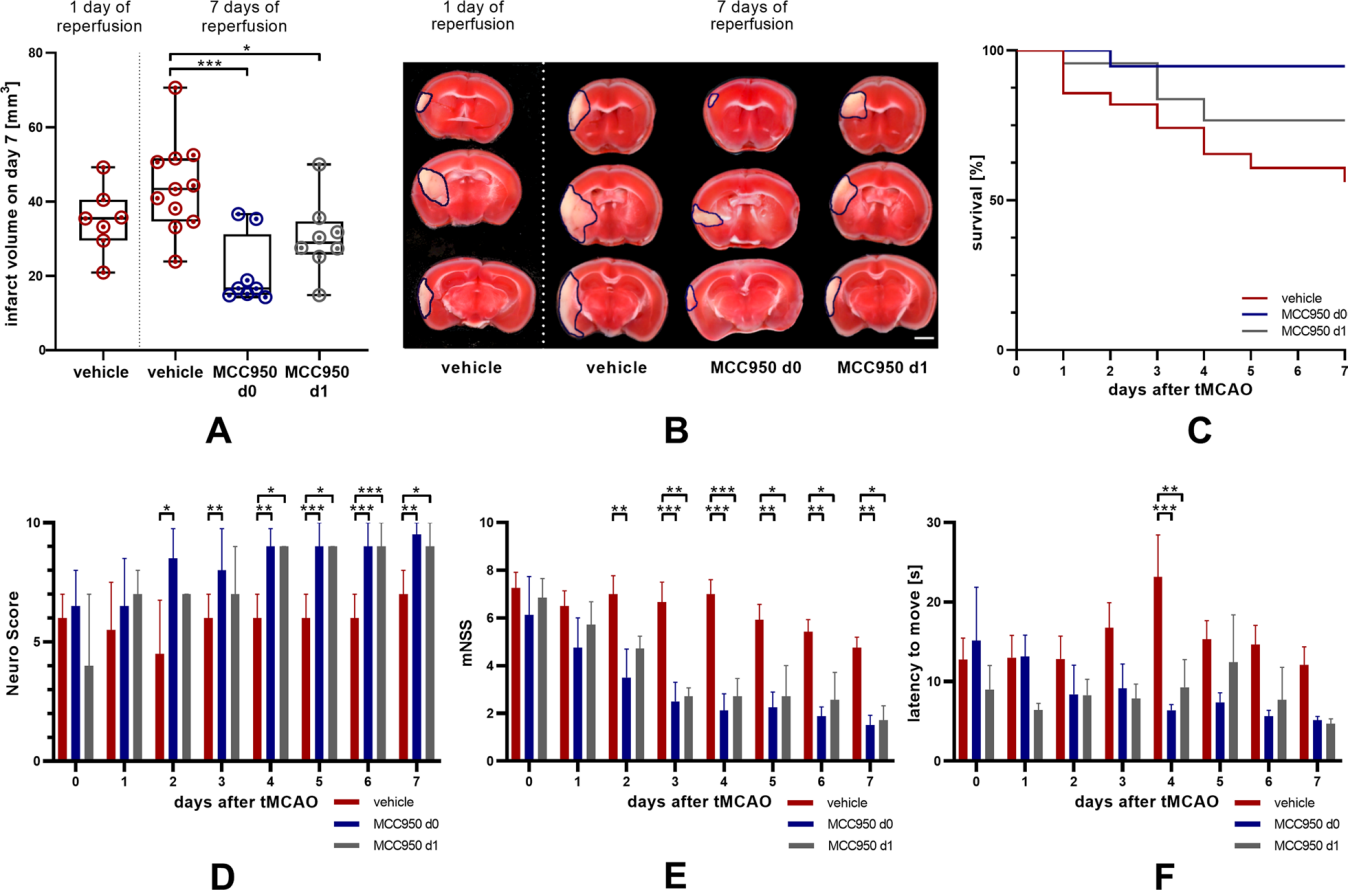
NLRP3 inhibition reduces stroke severity, secondary infarct growth, mortality and improves functional outcome as a long-term effect after prophylactic and delayed treatment 24 h after stroke onset. **A** Infarct volumetry (*n* ≥ 7) and **B** representative 2,3,5-triphenyltetrazolium chloride stainings of 3 consecutive coronal brain sections of vehicle-, MCC950 d0- and MCC950 d1-treated mice euthanized 7 days after tMCAO. In addition, infarct volumetry was performed 1 day after tMCAO of vehicle treated animals to visualize infarct progression between days 1 and 7. Scale bar = 2 mm. The infarcts are circled by a blue line. Data were analyzed by one-way ANOVA with post hoc Tukey adjustment for *p* values. *p < 0.05; ***p < 0.001. **C** Survival curve of vehicle-, MCC950 d0- and MCC950 d1-treated mice over a reperfusion period of 7 days. A log-rank test was performed to assess whether significant differences exist between the three study groups. Results show that survival distributions of the three interventions differ significantly, χ^2^ = 10.25, *p < 0.05. **D** The Neuro Score, **E** the modified Neurological Severity Scores (mNSS) and **F** the Latency To Move Test were performed on the day of tMCAO as well as daily during the 7 days of reperfusion (*n* ≥ 8). Data were analyzed by two-way ANOVA with post hoc Tukey adjustment for *p* values. *p < 0.05; **p < 0.01; ***p < 0.001

**Fig. 3:**
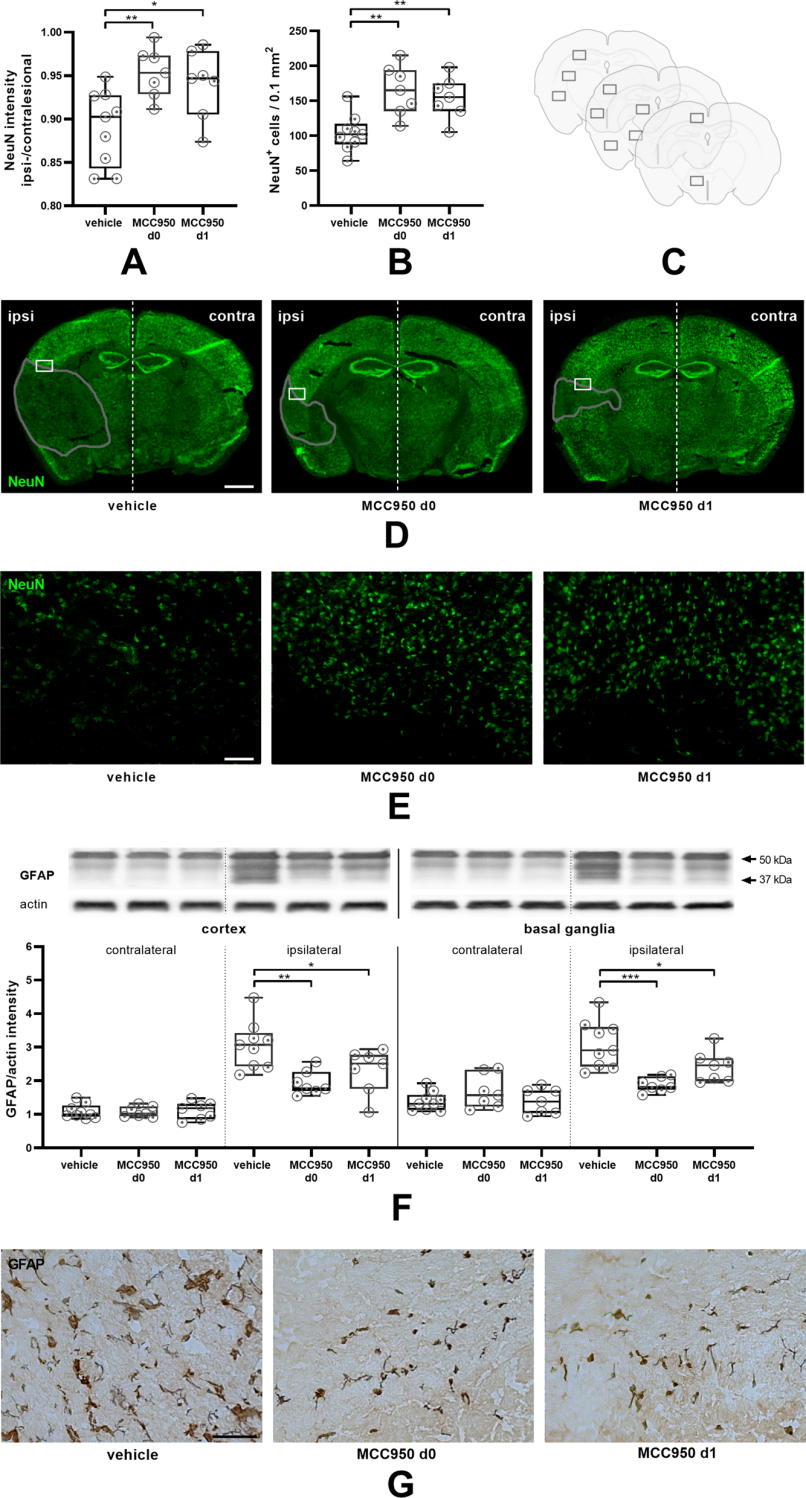
MCC950 has an enhanced long-term neuroprotective effect and reduces reactive astrogliosis after prophylactic and delayed application. **A** Ratio of ipsilesional to contralesional NeuN intensity of coronary brain slices 7 days after tMCAO of vehicle-, MCC950 d0- and MCC950 d1-treated mice. Three sections per animal. (*n* ≥ 7). **B** Number of NeuN positive cells per 0.1 mm^2^ counted in 10 corresponding regions on three sections per animal 7 days after tMCAO of vehicle-, MCC950 d0- and MCC950 d1-treated mice as indicated in (**C**). **D** Representative immunohistochemical stainings of NeuN (neurons, green) of coronary brain slices 7 d after tMCAO in vehicle-, MCC950 d0- and MCC950 d1-treated mice using 5 × objective. Scale bar = 2 mm. Infarct areas highlighted by a grey line. The white rectangles mark areas at the border of infarct core and penumbra as magnified 20-fold in (**E**). 20 × objective. Scale bar = 100 µm. **F** GFAP protein content in cortex or basal ganglia of vehicle-, MCC950 d0- and MCC950 d1-treated mice. For densitometric quantification actin was used as a loading control (*n* ≥ 7). **G** Representative penumbral stainings for GFAP of vehicle-, MCC950 d0- and MCC950 d1-treated mice. Scale bar = 50 µm. For densitometric quantification actin was used as a loading control. Data were analyzed by one-way ANOVA with post hoc Tukey adjustment for *p* values. *p < 0.05; **p < 0.01; ***p < 0.001

**Fig. 4:**
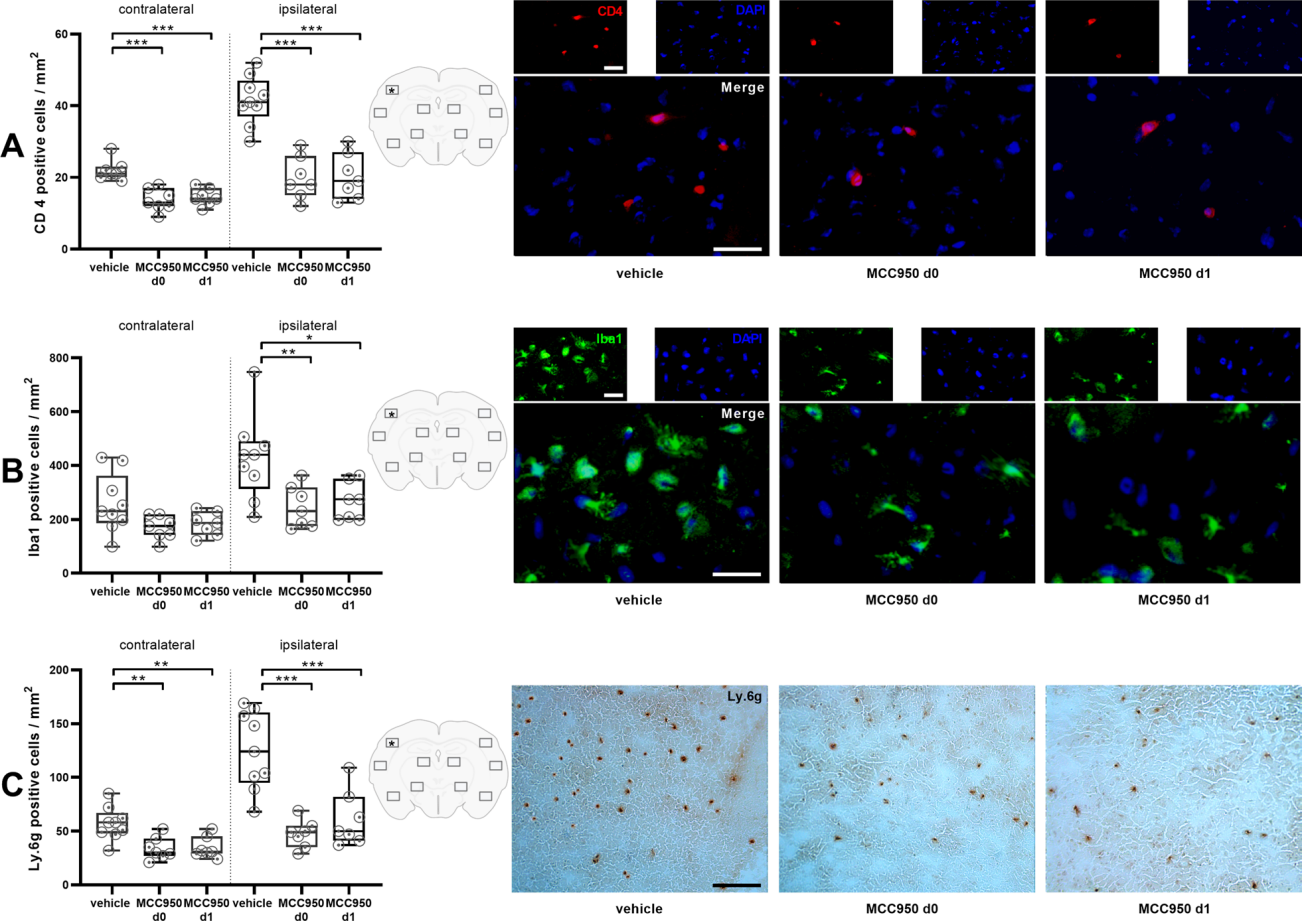
Prophylactic and delayed NLRP3 inhibition prevent long-range immune cell infiltration after IS. **A** Left: quantification of brain-infiltrating CD4-positive cells of vehicle-, MCC950 d0- and MCC950 d1-treated mice within 5 ipsilesional and 5 contralesional priorly defined segments as depicted in the center. Right: representative immunocytologic stainings (localization marked with a *) of CD4-positive lymphocytes (red) and nuclei (DAPI, blue) in the ipsilateral hemisphere on day 7 after tMCAO in vehicle-, MCC950 d0- and MCC950 d1-treated mice using 20 × objective. Scale bar = 20 μm (*n* ≥ 7). **B** Left: quantification of Iba1-positive cells of vehicle-, MCC950 d0- and MCC950 d1-treated mice within 5 ipsilesional and 5 contralesional priorly defined segments as depicted in the center. Right: representative immunocytologic stainings (localization marked with a *) of Iba1-positive microglia (green) and nuclei (DAPI, blue) in the ipsilateral hemisphere on day 7 after tMCAO in vehicle-, MCC950 d0- and MCC950 d1-treated mice using 20 × objective. Scale bar = 20 μm (*n* ≥ 7). **C** Left: quantification of brain-infiltrating Ly.6 g-positive cells of vehicle-, MCC950 d0- and MCC950 d1-treated treated mice within 5 ipsilesional and 5 contralesional priorly defined segments as depicted in the center. Right: representative stainings (localization marked with a *) of Ly.6 g-positive neutrophils in the ipsilateral hemisphere on day 7 after transient middle cerebral artery occlusion (tMCAO) in vehicle-, MCC950 d0- and MCC950 d1-treated mice using 10 × objective. Scale bar = 100 μm (*n* ≥ 7). Data were analyzed by one-way ANOVA with post hoc Tukey adjustment for *p* values. *p < 0.05, **p < 0.01; ***p < 0.001

We first assessed NLRP3 protein expression before and after MCAO. NLRP3 was still increased in mice 1 day after 30 min tMCAO in comparison with sham treated animals (Fig. 1). In a second step, we could observe that infarct volumes grow between day 1 and day 7 after 30 min tMCAO (Fig. 2A, B). We next analyzed the effect of delayed NLRP3 inflammasome inhibition on final infarct sizes at day 7 in mice that were subjected to 30 min tMCAO. Administration of MCC950 1 day after stroke induction (MCC950 d1) reduced stroke volumes by about 35% compared to controls (Fig. 2A, B, Additional file 1: Fig. S2). To determine the relative contribution of subacute parenchymal versus ultra-early intravascular inflammation to lesion evolution, we additionally treated mice prophylactically with MCC950 before tMCAO (MCC950 d0). As expected, prophylactic treatment, also covering the initial inflammation wave, reduced infarct volumes even more effective. These attenuated infarct sizes reflected in higher survival rates and better functional outcome; the latter assessed daily after tMCAO until day 7 (Fig. 2C–F). These improved outcomes of MCC950 d1 treated mice were accompanied by increased neuronal survival, reflected by a significantly higher proportion of ipsilesional to contralesional NeuN intensity (Fig. 3A), a higher number of surviving neurons (Fig. 3B–E) and reduced reactive astrogliosis, represented by glial fibrillary acidic protein (GFAP) WB measurements (Fig. 3F, G). Again, there is a trend showing superiority of prophylactic as compared to delayed treatment. These aforementioned therapeutic effects were associated with a modification of subacute intraparenchymal inflammation as shown by altered cytokine gene expression levels downstream to NLRP3 (Additional file 1: Fig. S3) and reduced immune cell infiltration. CD4-positive T-lymphocytes, Iba1-positive microglia as well as Ly6G-positive neutrophils were significantly reduced after MCC950 treatment without noteworthy difference in the treatment groups (Fig. 4, Additional file 1: Fig. S4). Altogether our data show for the first time that subacute stroke progression exists and that it is still amenable to NLRP3 inhibition.

## Discussion

As principal finding we show that infarct progression continues within the subacute stroke stage beyond 24 h after onset and is amenable to anti-inflammatory treatment by NLRP3 inflammasome inhibition. This is a novel and important finding, since it may open new avenues for modifying stroke outcome beyond recanalization, the only effective treatment to date in human stroke patients [27, 28]. It is well-established that the gene expression of NLRP3 and its downstream pro-inflammatory enzymes and cytokines, such as Caspase 1, IL1β or IL18, are upregulated in the ischemic hemisphere within the first hours after tMCAO in mice. The inhibition of NLRP3 alleviates this and leads to a better clinical outcome after 1 day of reperfusion by targeting I/R injury [11, 12, 14]. However, up to now, it has been unclear whether the second wave of inflammation, which occurs in the subacute stroke phase beyond 24 h after onset and which encompasses parenchymal leukocyte infiltration and glial activation, drives further infarct progression. We show that delayed NLRP3 inhibition after 1 day decreases secondary infarct growth. Reduced infarctions translated into improved behavioral outcome that we consider to be due to reduced neuronal damage (infarction), which is the consequence of targeting inflammatory processes with MCC950. Our results join a small series of earlier trials having shown an inflammation-driven infarct growth beyond 24 h after tMCAO: for instance a continuous infarct growth in mice between days 1 and 4 after tMCAO extending the classical timeframe of I/R injury. These observations were attributed to penumbral apoptotic cell death due to a detrimental role of T-cells [29–31]. It is not surprising that prophylactic treatment was even more effective, since it stabilizes the blood–brain barrier which is impaired early after vessel occlusion [12]. To our knowledge, however, it has never been shown before that the prophylactic inhibition of the NLRP3 inflammasome actually maintains its early treatment effect into the subacute phase.

Besides the strengths of our investigation, additional studies are necessary to finally evaluate the effects of NLRP3 inflammasome inhibition especially in the early phase (24–72 h) post stroke. One limitation is that we did not perform serial magnetic resonance imaging to analyze infarct dynamics, which would have enabled us to shed additional light on infarct development over time.

A recent study describing infarctions to reach their maximum size after 8 h of reperfusion does not contradict our findings, as they followed a 60 min tMCAO protocol resulting in much larger infarct volumes that comprised the whole anterior circulation [32]. Infarctions presented here, however, were much smaller in size (only “subletal”) leaving enough brain tissue to be damaged—or saved—secondarily. Even after 1 week of reperfusion the levels of inflammatory cells, namely, neutrophils, lymphocytes, microglia and monocytes, still remain significantly lower in the MCC950 treated groups.

Many studies have shown that NLRP3 plays an important role in inflammatory processes after ischemic stroke [11, 12, 14, 33–35]. However, two single studies found no evidence for an influence of this receptor on infarct growth. While one group used knock-out animals instead of our drug approach [36], the second group used MCC950, too, but a different stroke model (FeCl_3_) [37]. In our opinion, both knockout and FeCl_3_ models are less suitable for translation than our approach. Regardless, the conflicting results are evidence that further research into the inflammasome field in general and NLRP3 in particular is needed.

Taken together, we could show that the inhibition of the NLRP3 inflammasome in the acute phase of IS directly influences post stroke inflammation in the ischemic penumbra. Early treatment effects bear the propensity of a long-term immunomodulation, ultimately achieving a better neurologic outcome. Moreover, a secondary infarct growth in small/medium IS can be mitigated by NLRP3 inhibition. The underlying pathophysiology of the early and delayed infarct growth certainly differ from one another and need to be analyzed in future studies. The inflammasome-triggered final common path of neuroinflammation, though, is addressable by NLRP3 inhibition. With these new insights and given the efficacy of MCC950 in human probands ex vivo [38], MCC950 becomes an important candidate for translational research as additional acute stroke treatment regime with long-lasting prognosis-improving features.

## Supplementary Information

**Additional file 1: Table S1**. Primary antibody information. **Table S2.** Secondary antibody information. **Figure S1**. Survival Curve of the two vehicle groups. Before merging the two vehicle groups, the survival of the vehicle groups with prophylactic treatment (d0) and therapeutic treatment after 24 h of reperfusion (d1) was compared. A log-rank test was performed to assess whether significant differences exist between the two study groups. Results show that survival distributions of the two application times did not differ significantly, χ² = 0.02, p ~ 0.9. **Figure S2.** MAP2 staining to depict neuronal ischemic damage. NLRP3 inhibition reduces neuronal ischemic damage after prophylactic and delayed treatment 24h post stroke onset. (A) Infarct size comparison measured in percentage of the respective hemisphere (n ≥ 7) and (B) representative MAP2 stainings of vehicle-, MCC950 d0- and MCC950 d1-treated mice euthanized 7 days after tMCAO. Additionally, MAP2 staining was performed 1 day after tMCAO of vehicle treated animals to visualize infarct progression between days 1 and 7. Scale bar = 2 mm. The infarcts are circled by a blue line. Data was analyzed by 1-way ANOVA with post hoc Tukey adjustment for p values. *p < 0.05; ***p < 0.001. **Figure S3**. Cytokine gene expression downstream of NLRP3. Ipsilesional Caspase 1 and IL1b/18 gene expression levels are reduced by NLRP3 inhibition. Relative gene expression as detected by rtPCR of Caspase 1, interleukin-1β (IL1β) and interleukin-18 (IL18) (top) in the ischemic cortices and (bottom) basal ganglia of mice 7 d after 30 min transient middle cerebral artery occlusion (tMCAO) treated with either vehicle or MCC950 prophylactically (MCC d0)/ therapeutically (MCC950 d1) (n ≥ 5). **Figure S4**. Histological NLRP3-positivity of infiltrating immune cells. NLRP3 Co-staining of immune cells (A) Left: Percentage of NLRP3-positive cells within all CD4-positive cells of vehicle-, MCC950 d0- and MCC950 d1-treated mice within 5 ipsilesional priorly defined segments as in Figure 4. Right: Representative immunocytologic stainings (as depicted in red) of CD4-positive (red) as well as NLRP3-positive lymphocytes (green) and nuclei (DAPI, blue) in the ipsilateral hemisphere on day 7 after tMCAO in vehicle-, MCC950 d0- and MCC950 d1-treated mice using 20x objective. Scale bar = 75 μm (n ≥ 7). (B) Left: Percentage of NLRP3-positive cells within all Iba1-positive cells of vehicle-, MCC950 d0- and MCC950 d1-treated mice within 5 ipsilesional priorly defined segments as in Figure 4. Right: Representative immunocytologic stainings (as depicted in red) of Iba1-positive (red) as well as NLRP3-positive microglia (green) and nuclei (DAPI, blue) in the ipsilateral hemisphere on day 7 after tMCAO in vehicle-, MCC950 d0- and MCC950 d1-treated mice using 20x objective. Scale bar = 75 μm (n ≥ 7). (C) Left: Percentage of NLRP3-positive cells within all Ly.6g-positive cells of vehicle-, MCC950 d0- and MCC950 d1-treated mice within 5 priorly defined segments as in Figure 4. Right: Representative immunocytologic stainings (as depicted red) of Ly.6g-positive (red) as well as NLRP3-positive neutrophils (green) and nuclei (DAPI, blue) in the ipsilateral hemisphere on day 7 after tMCAO in vehicle-, MCC950 d0- and MCC950 d1-treated mice using 10x objective. Scale bar = 100 μm (n ≥ 7). Data was analyzed by 1-way ANOVA with post hoc Tukey adjustment for p values. *p < 0.05, **p < 0.01; ***p < 0.001. **Figure S5**. Iba1 Western Blot. Microglia Western Blot. Iba1 protein content in cortex or basal ganglia of vehicle- and sham-treated mice after 1 d and 7 d of reperfusion. For densitometric quantification actin was used as a loading control. (n ≥ 5). Data was analyzed by 1-way ANOVA with post hoc Tukey adjustment for p values. *p < 0.05, **p < 0.01. **Figure S6.** Negative control stainings. Figure S4: Negative controls of antibodies used for immunohistochemistry. Positive (first and second antibody) and negative (second antibody only) staining controls for (A) CD4 stainings, (B) Iba1 stainings, (C) Ly.6g stainings derived from spleen and (D) GFAP stainings derived from brain. 20 x magnification. (A-B) Scale bar = 50 µm, (B-C) Scale bar = 100 µm.

## Data Availability

The data sets used and/or analyzed during the current study are available from the corresponding author on reasonable request.

## Abbreviations

DAMPs: Danger-associated molecular patterns
GFAP: Glial fibrillary acidic protein
I/R: Ischemia/reperfusion
IgG: Immunoglobulin G
IL: Interleukin
IS: Ischemic stroke
mNSS: Modified Neurological Severity Score
NLRP3 inflammasome: NLR-family pyrin domain containing 3 inflammasome
PBS: Phosphate buffered saline
tMCAO: Transient middle cerebral artery occlusion
