# Supplementary material for: Delayed NLRP3 inflammasome inhibition ameliorates subacute stroke progression in mice
Source: J Neuroinflammation. 2023 Jan 4;20:4. doi: 10.1186/s12974-022-02674-w (PMC9811791; doi:10.1186/s12974-022-02674-w)
Supplement: Supplementary file 1 — Additional file 1: Table S1. Primary antibody information. Table S2. Secondary antibody information. Figure S1. Survival Curve of the two vehicle groups. Before merging the two vehicle groups, the survival of the vehicle groups with prophylactic treatment (d0) and therapeutic treatment after 24 h of reperfusion (d1) was compared. A log-rank test was performed to assess whether significant differences exist between the two study groups. Results show that survival distributions of the two application times did not differ significantly, χ² = 0.02, p ~ 0.9. Figure S2. MAP2 staining to depict neuronal ischemic damage. NLRP3 inhibition reduces neuronal ischemic damage after prophylactic and delayed treatment 24h post stroke onset. (A) Infarct size comparison measured in percentage of the respective hemisphere (n ≥ 7) and (B) representative MAP2 stainings of vehicle-, MCC950 d0- and MCC950 d1-treated mice euthanized 7 days after tMCAO. Additionally, MAP2 staining was performed 1 day after tMCAO of vehicle treated animals to visualize infarct progression between days 1 and 7. Scale bar = 2 mm. The infarcts are circled by a blue line. Data was analyzed by 1-way ANOVA with post hoc Tukey adjustment for p values. *p < 0.05; ***p < 0.001. Figure S3. Cytokine gene expression downstream of NLRP3. Ipsilesional Caspase 1 and IL1b/18 gene expression levels are reduced by NLRP3 inhibition. Relative gene expression as detected by rtPCR of Caspase 1, interleukin-1β (IL1β) and interleukin-18 (IL18) (top) in the ischemic cortices and (bottom) basal ganglia of mice 7 d after 30 min transient middle cerebral artery occlusion (tMCAO) treated with either vehicle or MCC950 prophylactically (MCC d0)/ therapeutically (MCC950 d1) (n ≥ 5). Figure S4. Histological NLRP3-positivity of infiltrating immune cells. NLRP3 Co-staining of immune cells (A) Left: Percentage of NLRP3-positive cells within all CD4-positive cells of vehicle-, MCC950 d0- and MCC950 d1-treated mice within 5 ipsilesional priorly [file 12974_2022_2674_MOESM1_ESM.pdf]

Additional Material

Table S1: Primary antibody information

antibody name	catalogue #	supplier	host
anti-β-Actin	A5441	Merck, Darmstadt, Germany	mouse
anti-CD4	100506	BioLegend, San Diego, CA, USA	rat
anti-GFAP	ab7260	Abcam, Cambridge, UK	rabbit
anti-Iba1	ab178846	Abcam, Cambridge, UK	rabbit
anti-Ly6G	127636	BioLegend, San Diego, CA, USA	rat
anti-NeuN	MAB377	Merck, Darmstadt, Germany	mouse
anti-NLRP3	AG-20B-0014	Adipogen Life Sciences, San Diego, CA, USA	mouse
anti-MAP2	ab32454	Abcam, Cambridge, UK	rabbit
Peroxidase AffiniPure IgG	715-035-150	Jackson ImmunoResearch, Cambridge, UK	donkey

Table S2: Secondary antibody information

antibody name	catalogue #	supplier	host / target
Alexa FluorTM 488 IgG	A11001	Thermo Fisher Scientific, Waltham, MA, USA	goat anti-mouse
Alexa FluorTM 488 IgG	A21206	Thermo Fisher Scientific, Waltham, MA, USA	donkey anti-rabbit
Alexa FluorTM 488 IgG	A21208	Thermo Fisher Scientific, Waltham, MA, USA	donkey anti-rat
Alexa FluorTM 546 IgG	A11035	Thermo Fisher Scientific, Waltham, MA, USA	goat anti-rabbit
Alexa FluorTM 647 IgG	A21247	Thermo Fisher Scientific, Waltham, MA, USA	goat anti-rat
Peroxidase AffiniPure Goat Anti-Rabbit IgG	111-035-003	Jackson ImmunoResearch, Cambridge, UK	goat anti-rabbit
Peroxidase AffiniPure IgG	715-035-150	Jackson ImmunoResearch, Cambridge, UK	donkey anti-mouse

Figure S1: Survival Curve of the two vehicle groups

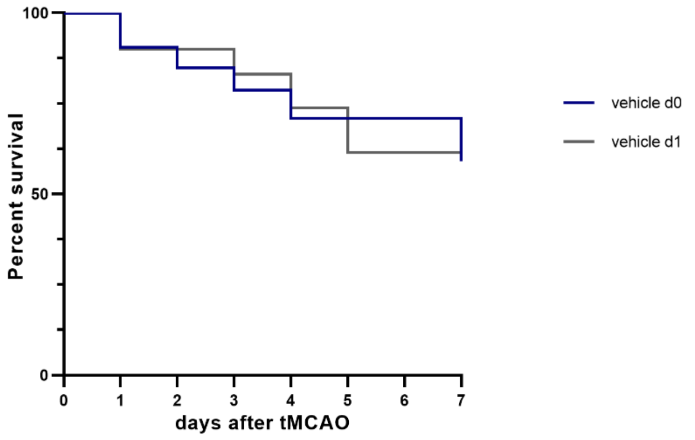

Figure S1: Before merging the two vehicle groups, the survival of the vehicle groups with prophylactic treatment (d0) and therapeutic treatment after 24 h of reperfusion (d1) was compared. A log-rank test was performed to assess whether significant differences exist between the two study groups. Results show that survival distributions of the two application times did not differ significantly, $\chi^2 = 0.02$, $p \sim 0.9$.

Figure S2: MAP2 staining to depict neuronal ischemic damage

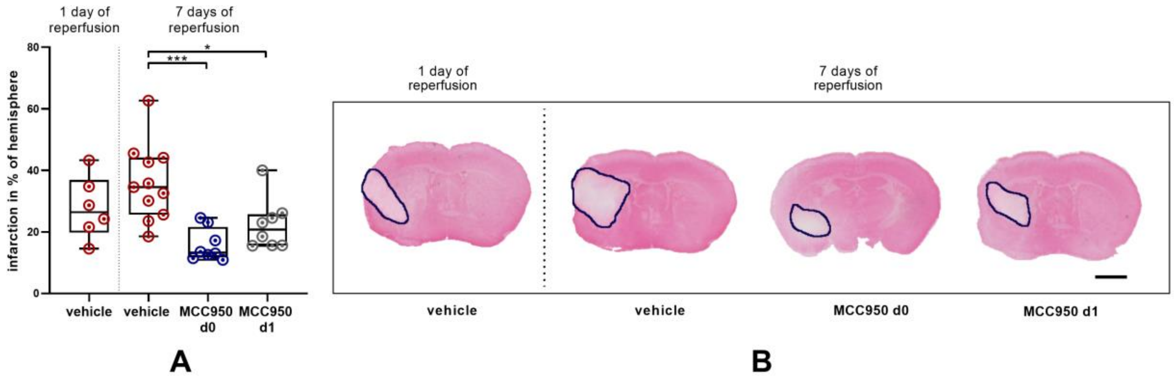

Figure S2: **NLRP3 inhibition reduces neuronal ischemic damage after prophylactic and delayed treatment 24h post stroke onset**. (A) Infarct size comparison measured in percentage of the respective hemisphere (n ≥ 7) and (B) representative MAP2 stainings of vehicle-, MCC950 d0- and MCC950 d1-treated mice euthanized 7 days after tMCAO. Additionally, MAP2 staining was performed 1 day after tMCAO of vehicle treated animals to visualize infarct progression between days 1 and 7. Scale bar = 2 mm. The infarcts are circled by a blue line. Data was analyzed by 1-way ANOVA with post hoc Tukey adjustment for p values. $*p < 0.05$; $***p < 0.001$.

Figure S3: Cytokine gene expression downstream of NLRP3

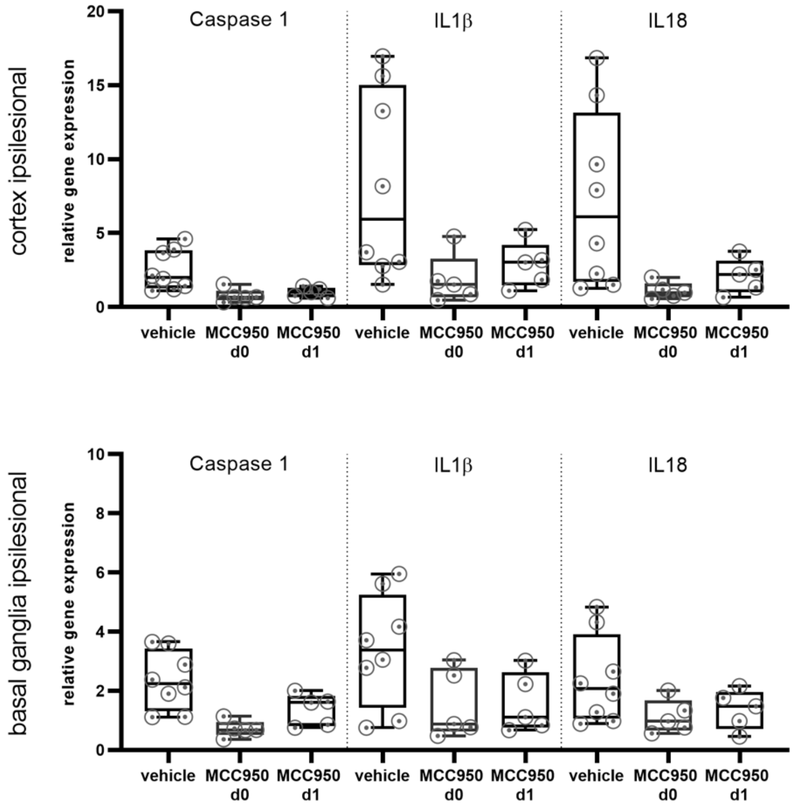

Figure S3: Ipsilesional Caspase 1 and IL1β/18 gene expression levels are reduced by NLRP3 inhibition. Relative gene expression as detected by rtPCR of Caspase 1, interleukin-1β (IL1β) and interleukin-18 (IL18) (top) in the ischemic cortices and (bottom) basal ganglia of mice 7 d after 30 min transient middle cerebral artery occlusion (tMCAO) treated with either vehicle or MCC950 prophylactically (MCC d0)/ therapeutically (MCC950 d1) (n ≥ 5).

Figure S4: Histological NLRP3-positivity of infiltrating immune cells

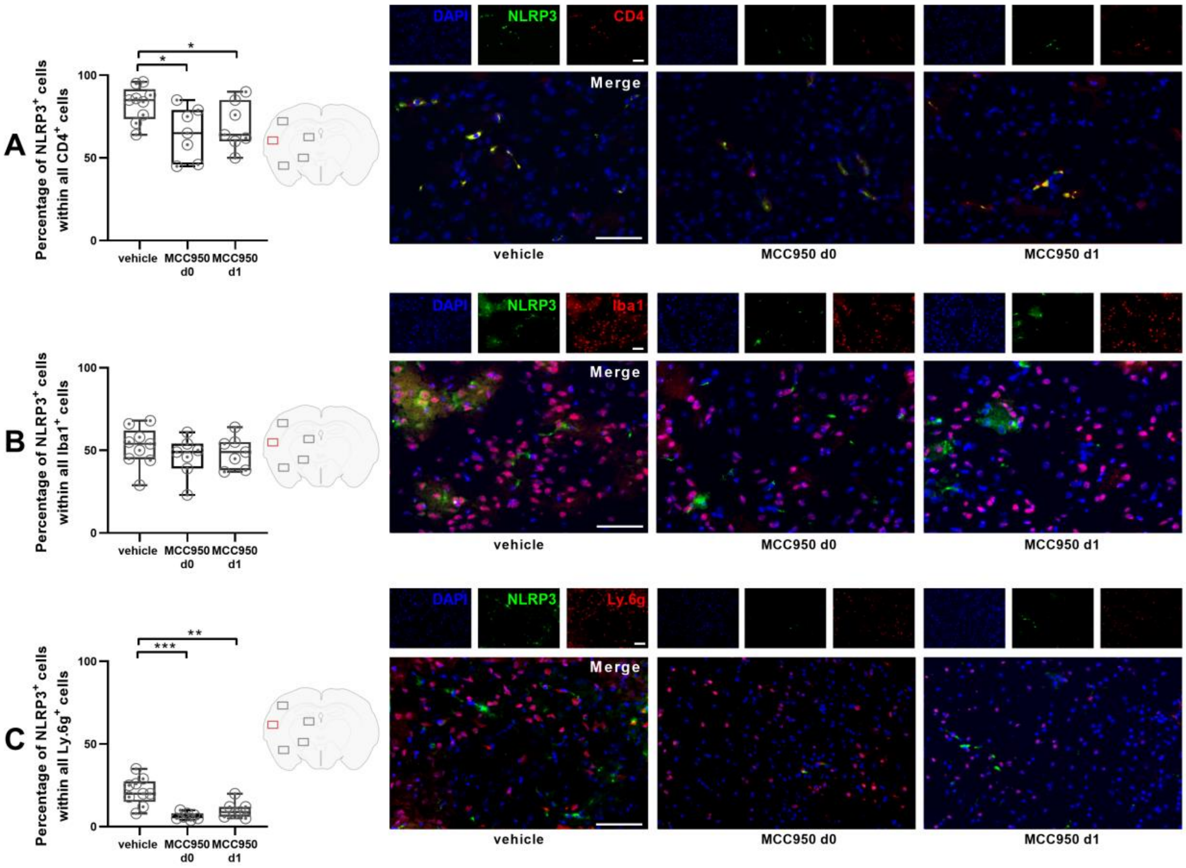

Figure S4: NLRP3 Co-staining of immune cells (A) Left: Percentage of NLRP3-positive cells within all CD4-positive cells of vehicle-, MCC950 d0- and MCC950 d1-treated mice within 5 ipsilesional priorly defined segments. Right: Representative immunocytologic stainings (as depicted in red) of CD4-positive (red) as well as NLRP3-positive (green) lymphocytes and nuclei (DAPI, blue) in the ipsilateral hemisphere on day 7 after tMCAO in vehicle-, MCC950 d0- and MCC950 d1-treated mice using 20x objective. Scale bar = 75 µm (n ≥ 7). (B) Left: Percentage of NLRP3-positive cells within all Iba1-positive cells of vehicle-, MCC950 d0- and MCC950 d1-treated mice within 5 ipsilesional priorly defined segments. Right: Representative immunocytologic stainings (as depicted in red) of Iba1-positive (red) as well as NLRP3-positive (green) microglia and nuclei (DAPI, blue) in the ipsilateral hemisphere on day 7 after tMCAO in vehicle-, MCC950 d0- and MCC950 d1-treated mice using 20x objective. Scale bar = 75 µm (n ≥ 7). (C) Left: Percentage of NLRP3-positive cells within all Ly.6g-positive cells of vehicle-, MCC950 d0- and MCC950 d1-treated mice within 5 priorly defined segments. Right: Representative immunocytologic stainings (as depicted red) of Ly.6g-positive (red) as well as NLRP3-positive (green) neutrophils and nuclei (DAPI, blue) in the ipsilateral hemisphere on day 7 after tMCAO in vehicle-, MCC950 d0- and MCC950 d1-treated mice using 10x objective. Scale bar = 100 µm (n ≥ 7). Data was analyzed by 1-way ANOVA with post hoc Tukey adjustment for p values. *p < 0.05, **p < 0.01; ***p < 0.001.

Figure S5: Iba1 Western Blot

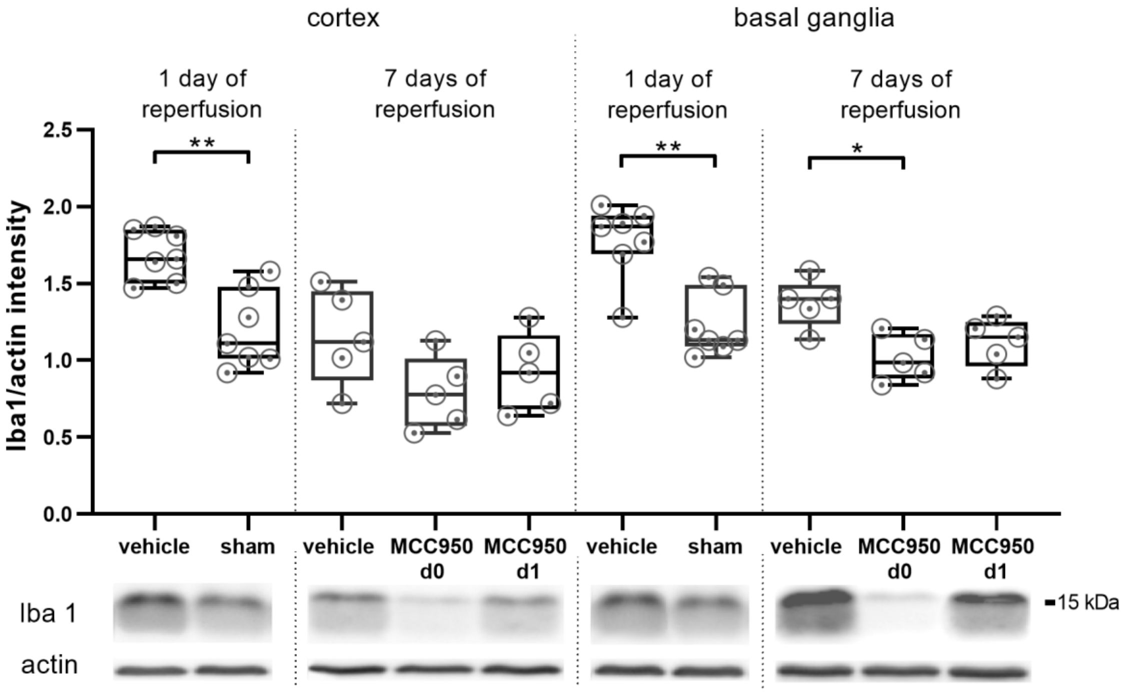

Figure S5: Microglia Western Blot. Iba1 protein content in cortex or basal ganglia of vehicle- and sham-treated mice after 1 d and 7 d of reperfusion. For densitometric quantification actin was used as a loading control. (n ≥ 5). Data was analyzed by 1-way ANOVA with post hoc Tukey adjustment for p values. *p < 0.05, **p < 0.01.

Figure S6: Negative control stainings

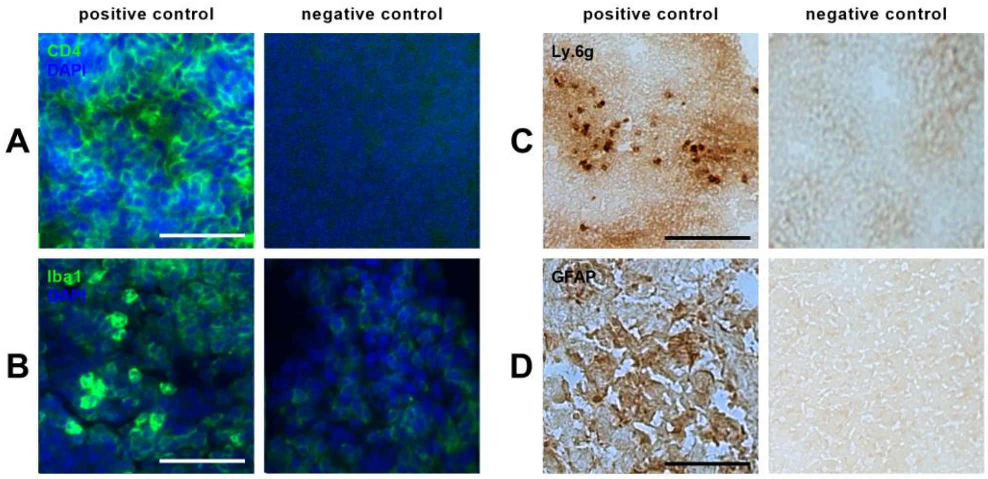

Figure S4: Negative controls of antibodies used for immunohistochemistry. Positive (first and second antibody) and negative (second antibody only) staining controls for (A) CD4 stainings, (B) Iba1 stainings, (C) Ly.6g stainings derived from spleen and (D) GFAP stainings derived from brain. 20 x magnification. (A-B) Scale bar = 50 µm, (B-C) Scale bar = 100 µm.
